# Effects of dietary fiber content and different fiber-rich ingredients on endogenous loss of fat and fatty acids in growing pigs

**DOI:** 10.1186/s40104-019-0348-3

**Published:** 2019-06-13

**Authors:** Yifan Chen, Zhenyu Wang, Jian Ding, Dongxu Ming, Wenhui Wang, Zhaoning Jiang, Ling Liu, Fenglai Wang

**Affiliations:** 1 State Key Laboratory of Animal Nutrition, China Agricultural University, No. 2 Yuanmingyuan West Road, Beijing, 100193 China; 2 National Animal Husbandry Service, Building No. 20, Maizidian street, Chaoyang District, Beijing, 100125 China

**Keywords:** Endogenous loss, Fat, Fatty acids, Fiber content, Fiber-rich ingredients, Growing pigs

## Abstract

**Background:**

Determination of the endogenous loss of fat (ELF) is used to adjust for the estimation of true total tract digestibility (TTTD) of fat in diets and ingredients. Any factor which affected ELF may further affect the digestibility of fat, including sources and concentrations of fat and fiber in the diet. There are some reports of determining the ELF using regression methods based on different levels of fat intake, while reports on effects of dietary fiber content and different fiber-rich ingredients in pig diets on ELF are very limited. Therefore, the objective of this study was to determine the effects of dietary fiber content and different fiber-rich ingredients on endogenous losses of fat and fatty acids at the end of ileum and throughout the entire intestinal tract in growing pigs.

**Methods:**

In Exp. 1, the effect of fiber content on endogenous loss of fat was determined using six growing pigs (Duroc × Landrace × Yorkshire; 27.6 ± 2.4 kg), fitted with a T-cannula at the end of ileum. The experimental design was a 6 × 6 complete Latin square design with six periods of feeding and six diets. The six experimental fat-free diets were formulated to include graded levels of neutral detergent fiber (NDF) (0, 40, 80, 120, 160 and 200 g/kg) and soybean hull (SH) was the only fiber source, providing 0, 75, 150, 225, 300 and 375 g/kg, respectively. Chromic oxide was included at 4 g/kg in all diets as an indigestible marker. In Exp. 2, six crossbred growing barrows (27.6 ± 1.6 kg) were used and the experimental design was the same as for Exp. 1. The six fat-free diets were formulated to include six common fiber-rich ingredients and the concentration of NDF was 100 g/kg. The six fiber-rich ingredients were defatted rice bran (DRB), sugar beet pulp (SBP), rice hull (RH), corn germ meal (CGM), SH and wheat bran (WB) and they were fed at represented 250, 270, 145, 250, 170 and 280 g/kg in the diet, respectively.

**Results:**

In Exp. 1, the endogenous loss of fatty acids profile did not change as dietary NDF increased in growing pigs. The endogenous losses of fat, C16:0, C18:0, C18:1, C18:2, total unsaturated fatty acids (UFA) and total saturated fatty acids (SFA) in growing pigs at the end of ileum and throughout the entire intestinal tract increased linearly as NDF content of diets increased. The endogenous losses of fat, as well as C16:0 and C18:0 throughout the entire intestinal tract also increased quadratically as NDF content of diets increased. The ELF increased from 0.71 to 3.14 g/kg of dry matter intake (DMI) and 0.56 to 8.21 g /kg DMI at the end of ileum and throughout the entire intestinal tract in growing pigs, respectively. The ELF occurred in the hindgut except for the growing pigs fed 0 and 4% NDF in their diets. The endogenous losses of C16:0 and UFA occurred primarily in the upper regions of the gut and the greatest endogenous losses of C18:0 occurred in the hindgut. The endogenous losses of fat, individual SFA and total SFA throughout the entire intestinal tract were much greater than that at the end of ileum. However, the endogenous losses of individual UFA and total UFA were less throughout the intestinal tract than at the end of ileum. In Exp. 2, the endogenous losses of fat at the end of ileum were greater in growing pigs fed CGM or WB diets. The endogenous loss of fatty acids profile changed to a slight degree at the end of ileum that the endogenous loss of UFA (particularly C18:1 and C18:2) in growing pigs fed CGM or WB diets were greater (*P* <  0.01) than that for the other four diets. The greatest (*P* <  0.01) endogenous loss of SFA (particularly C18:0) was in growing pigs fed the RH diet. The endogenous losses of fat, C16:0, C18:0 and SFA over the entire intestinal tract were much greater in growing pigs fed CGM or WB diets, whereas the lowest values were in growing pigs fed DRB diet. The ELF at the end of ileum in growing pigs fed CGM or WB diets were 3.50 or 4.17 g/kg DMI, respectively, and the ELF over the entire intestinal tract was 7.23 or 7.38 g/kg DMI. The contribution in percentage of ELF in the upper gut was greater than that in the hindgut of growing pigs fed DRB and RH diets, while the ELF in the upper gut and hindgut were equal in growing pigs fed SBP, CGM and WB diets. On the whole, the endogenous losses of C18:1 and C18:2 throughout the entire intestinal tract in growing pigs fed the six fiber-rich ingredients diets were less than losses at the end of ileum, whereas the endogenous loss of fat, C16:0, C18:0 and SFA were greater throughout the intestinal tract than at the end of ileum.

**Conclusion:**

The profile of loss in endogenous fatty acids did not change as dietary NDF increased in growing pigs and the endogenous losses of fatty acids (C16:0, C18:0, C18:1 and C18:2) fat, UFA and SFA increased linearly as NDF content increased in the diets of pigs. The endogenous losses of fat or fatty acids at the end of ileum were greater in growing pigs fed RH, CGM or WB diets. The endogenous losses of fat, fatty acids (C16:0 and C18:0) and SFA were greater over the entire intestinal tract in pigs fed CGM or WB diet, while these values were the lowest in growing pigs fed the DRB diet. The contribution in percentage losses of fat in the upper gut were greater than in the hindgut of growing pigs fed DRB and RH diets, while the contribution of losses of fat in the upper gut and hindgut were equal in growing pigs fed SBP, CGM and WB diets. In addition, the endogenous loss of individual or total UFA was less over the entire intestinal tract of growing pigs fed fiber diets than that at the end of ileum, and the greatest endogenous losses of fat, individual or total SFA were over the entire intestinal tract. Therefore, differences in fiber content and the nature of fiber-rich ingredients in diets of pigs have different effects to the endogenous losses of fat or fatty acids. Considering the requirement of fat or fatty acids of pigs, careful attention must be paid that the endogenous losses of fat and fatty acids when fiber ingredients are used in diets of pigs.

## Background

Endogenous loss of fat (ELF) occurs during digestion along the intestinal tract and the unabsorbed portion of the endogenous fat is excreted into the gastrointestinal tract originating from bile, mucosal exudation, cholesterol or structural lipids from desquamation of epithelial cells and the *de novo* synthesis of fatty acids by the microbial population [1, 2]. Endogenous loss of fat may lead to complications in determining the digestibility of fat and fatty acids. Some reports indicate that dietary fat intake affects the apparent total tract digestibility (ATTD) of fat which would be expected to increase as fat intake increases [3–6]. This phenomenon may result from a decrease in the ratio between ELF and fat intake in animals fed high fat diets, reflecting a lower ATTD of fat in low fat diets instead of an increase in the digestibility of fat in high fat diets [7–10]. However, the true total tract digestibility (TTTD) of fat is not affected by the intake of dietary fat [9, 11, 12]. That conclusion is based on results of ELF measurements that allow adjustments in the estimation of TTTD of fat in diets and their ingredients [9]. Therefore, the accurate determination of ELF is essential, but may not, in some instances, of concern when considering diets of pigs.

Estimates of ELF in pigs have been reported, but the values have been not consistent [3, 7, 8, 11, 13]. The inconsistencies in reported ELF values are closely related to many factors. Any factor which affect the values of ELF may further affect the digestibility of fat, including the sources and dietary concentrations of fat and fiber [6, 14, 15]. Some studies have reported ELF values using the regression method based on different levels of fat intake [3, 7, 8, 11, 13]. In particular, Kil et al. [11] reported that the ELF value of extracted fat was lower than that for intact fat, which was correlated with the fiber of intact fat. Nevertheless, studies correlating effects of fiber content and different fiber-rich ingredients in diets of pigs on ELF are very limited.

In addition, the ELF in pigs has been evaluated over the entire intestinal tract, while information on the ELF at the end of ileum is limited. Furthermore, the determination of effect of fiber on ELF is necessary for guidance on adding proper quantities of lipids or specific fatty acids in diets of pigs to maintain an acceptable level of dietary energy and meet requirements for fatty acids in pigs fed diets including fiber-rich ingredients. Therefore, the aim of this study was to evaluate the effect of fiber content and different fiber-rich ingredients on losses of endogenous fat and fatty acids at the end of ileum and over the entire intestinal tract in growing pigs.

## Methods

### Animal, dietary treatments and experimental design

Two experiments were performed in the Metabolism Laboratory of the Ministry of Agriculture Feed Industry Centre (China Agricultural University, Beijing, China). All diets in the two experiments were fed in mash form. The experimental pigs (Duroc × Landrace × Yorkshire) were obtained from the Swine Nutrition Research Centre of the National Feed Engineering Technology Research Centre (Chengde, China).

Exp. 1 was designed to evaluate the effect of fiber content on losses of endogenous fat and fatty acids at the end of ileum and over the entire intestinal tract in growing pigs. Six crossed growing pigs (Duroc × Landrace × Yorkshire, initial BW = 27.6 ± 2.4 kg) were housed individually in stainless-steel metabolism crates (1.4 m × 0.7 m × 0.6 m), located in a temperature-controlled room (22 ± 2 °C). A self-feeder and a low-pressure nipple drinker were provided in each metabolism crates. All pigs were surgically fitted with a T-cannula at the end of ileum according as described by Stein et al. [16]. After a 14-day recovery from surgery, initial BW of pigs was recorded and then pigs were assigned randomly to one of six dietary treatments in a 6-period complete Latin square design. There were 6 observations per treatment. Pigs were fed a grower diet (160 g/kg crude protein, CP) during the 14 d post-surgery period. The amount of feed was increased by 100 g daily until feed intake reached a level of 4% of BW. The BW of the pigs was obtained at the beginning of each period and then the feed intake was adjusted accordingly for each pig. Two equal-sized meals were supplied at 08:00 and 17:00 h daily. Water was provided *ad libitum*.

The compositions of the dietary treatments in Exp. 1 are shown in Table 1. The six experimental diets were formulated to include 0, 40, 80, 120, 160 and 200 g/kg of neutral detergent fiber (NDF). Soybean hull (SH), the only fiber source, was provided at 0, 75, 150, 225, 300 or 375 g/kg in the same respective diets, respectively. Chromic oxide was included at 4 g/kg in each diet as an indigestible marker. Vitamins and minerals were provided in each diet to meet or exceed the nutrient requirements for growing pigs as recommended by the National Research Council (NRC) [17]. The analyzed composition of diets in Exp. 1 are summarized in Table 2.

**Table 1:** Composition of experimental diets in Exp. 1 (as-fed basis)

Items, g/kg	Neutral detergent fiber content of diets, g/kg					
	0	40	80	120	160	200
Corn starch	649.3	598.1	547.0	495.8	444.7	393.5
Casein	198.3	182.7	167.0	151.5	135.8	120.2
Soybean hull	0.0	75.0	150.0	225.0	300.0	375.0
Sucrose	104.4	96.2	88.0	79.7	71.5	63.3
Dicalcium phosphate	25.0	25.0	25.0	25.0	25.0	25.0
Limestone	5.0	5.0	5.0	5.0	5.0	5.0
Salt	4.0	4.0	4.0	4.0	4.0	4.0
Potassium carbonate	4.0	4.0	4.0	4.0	4.0	4.0
Magnesium oxide	1.0	1.0	1.0	1.0	1.0	1.0
Chromic oxide	4.0	4.0	4.0	4.0	4.0	4.0
Mineral and vitamin premix^a^	5.0	5.0	5.0	5.0	5.0	5.0
Total	1,000	1,000	1,000	1,000	1,000	1,000

^a^Vitamin-mineral premix supplied the following nutrients per kilogram of diet: vitamin A, 5512 IU; vitamin D_3_, 2200 IU; vitamin E, 30 IU; vitamin K_3_, 2.2 mg; vitamin B_12_, 27.6 μg; riboflavin, 4 mg; pantothenic acid, 14 mg; niacin, 30 mg; choline chloride, 400 mg; folic acid, 0.7 mg; thiamine, 1.5 mg; pyridoxine, 3 mg; biotin, 44 μg; Mn (MnO), 40 mg; Fe (FeSO_4_·H_2_O), 75 mg; Zn (ZnO), 75 mg; Cu (CuSO_4_·5H_2_O), 100 mg; I (KI), 0.3 mg; Se (Na_2_SeO_3_), 0.3 mg

**Table 2:** Analyzed composition of experimental diets in Exp. 1

Items, g/kg^a^	NDF content of diets, g/kg					
	0	40	80	120	160	200
DM	913.5	911.8	911.2	913.6	913.0	914.4
EE	2.17	4.81	6.09	8.34	11.55	12.51
C12:0	0.39	0.43	0.43	0.52	0.45	0.29
C14:0	0.53	0.55	0.51	0.46	0.43	0.42
C16:0	2.94	3.34	3.7	4.09	4.31	4.86
C18:0	0.89	1.06	1.24	1.44	1.57	1.78
C18:1	1.56	2.2	2.97	3.71	4.66	5.57
C18:2	1.92	3.07	4.78	6.39	8.29	10.62
C18:3	0.15	0.36	0.66	0.49	1.28	1.71
C20:0	0.03	0.05	0.09	0.09	0.10	0.12
C20:1	0.01	0.02	0.02	0.04	0.04	0.05
C22:0	0.04	0.05	0.00	0.01	0.01	0.02
C24:0	0.05	0.06	0.07	0.08	0.09	0.11
SFA	4.94	5.63	6.21	6.86	7.14	7.82
UFA	3.64	5.65	8.44	10.62	14.27	17.94
CP	169.8	172.0	168.9	165.4	166.0	164.6
SDF	14.5	12.5	12.0	16.5	12.5	22.0
IDF	9.0	51.5	95.5	144.0	186.0	237.5
TDF	22.5	63.5	107	160.5	198.5	259.5
NDF	8.9	51.1	95.1	134.0	188.9	218.9
ADF	1.9	32.3	62.2	90.6	126.9	148.0
Hemicellulose	7.0	18.8	32.9	43.4	62.0	70.9

^a^*DM* dry matter, *EE* ether extract, *SFA* saturated fatty acid, *UFA* unsaturated fatty acid, *CP* crude protein, *SDF* soluble dietary fiber, *IDF* insoluble dietary fiber, *TDF* total dietary fiber, *NDF* neutral detergent fiber, *ADF* acid detergent fiber

Exp. 2 was conducted to determine the effect of different fiber-rich ingredients on the endogenous fat and fatty acids losses at the end of ileum and over the entire intestinal tract in growing pigs. Six crossbred growing barrows (Duroc × Landrace × Yorkshire), initial weighing 27.6 ± 1.6 kg, were used. The experimental design was as described for Exp. 1. The compositions of the dietary treatments are summarized in Table 3. The six experimental diets were formulated to include six different fiber-rich ingredients containing little fat and NDF concentrations were about 100 g/kg across the diets. The six fiber-rich ingredients were defatted rice bran (DRB), sugar beet pulp (SBP), rice hull (RH), corn germ meal (CGM), SH and wheat bran (WB) provided as 250, 270, 145, 250, 170 and 280 g/kg, respectively. Chromic oxide was supplemented at 4 g/kg in each diet as an indigestible marker. Vitamins and minerals were included in each diet to meet or exceed the nutrient requirements for growing pigs recommended by NRC (2012) [17]. The analyzed composition of the experimental diets are summarized in Table 4.

**Table 3:** Composition of experimental diets in Exp. 2 (as-fed basis)

Items, g/kg	Fiber-rich ingredients of diets^b^					
	250 g/kg DRB	270 g/kg SBP	145 g/kg RH	250 g/kg CGM	170 g/kg SH	280 g/kg WB
Corn Starch	477	446	544	490	542	463
Casein	125	136	163	112	140	109
DRB	250	–	–	–	–	–
SBP	–	270	–	–	–	–
RH	–	–	145	–	–	–
CGM	–	–	–	250	–	–
SH	–	–	–	–	170	–
WB	–	–	–	–	–	280
Sucrose	100	100	100	100	100	100
Dicalcium phosphate	25	25	25	25	25	25
Limestone	5	5	5	5	5	5
Salt	4	4	4	4	4	4
Potassium carbonate	4	4	4	4	4	4
Magnesium oxide	1	1	1	1	1	1
Chromic oxide	4	4	4	4	4	4
Mineral and vitamin premix^a^	5	5	5	5	5	5
Total	1000	1000	1000	1000	1000	1000

^a^Vitamin-mineral premix supplied the following nutrients per kilogram of diet: vitamin A, 5512 IU; vitamin D_3_, 2200 IU; vitamin E, 30 IU; vitamin K_3_, 2.2 mg; vitamin B_12_, 27.6 μg; riboflavin, 4 mg; pantothenic acid, 14 mg; niacin, 30 mg; choline chloride, 400 mg; folic acid, 0.7 mg; thiamine, 1.5 mg; pyridoxine, 3 mg; biotin, 44 μg; Mn (MnO), 40 mg; Fe (FeSO_4_·H_2_O), 75 mg; Zn (ZnO), 75 mg; Cu (CuSO_4_·5H_2_O), 100 mg; I (KI), 0.3 mg; Se (Na_2_SeO_3_), 0.3 mg

^b^ *DRB* defatted rice bran, *SBP* sugar beet pulp, *RH* rice hull, *CGM* corn germ meal, *SH* soybean hull, *WB* wheat bran

**Table 4:** Analyzed composition of experimental diets in Exp. 2

Items, g/kg^b^	Fiber-rich ingredients of diets^a^					
	250 g/kg DRB	270 g/kg SBP	145 g/kg RH	250 g/kg CGM	170 g/kg SH	280 g/kg WB
DM	910.8	909.9	914.2	912.5	910.3	910.3
EE	3.29	1.39	0.51	1.14	6.27	9.33
C12:0	0.37	0.59	0.60	0.35	0.53	0.57
C14:0	0.43	0.42	0.49	0.37	0.41	0.33
C16:0	3.15	3.13	2.72	3.61	3.57	5.30
C18:0	0.80	0.71	0.85	0.76	1.21	0.79
C18:1	2.24	1.5	1.43	3.18	2.91	3.86
C18:2	2.57	2.72	1.65	5.50	5.06	8.63
C18:3	0.18	0.16	0.09	0.27	0.72	0.57
C20:0	0.05	0.04	0.04	0.07	0.07	0.06
C20:1	0.02	0.03	0.02	0.03	0.03	0.15
C22:0	0.05	0.05	0.04	0.08	0.00	0.04
C24:0	0.08	0.09	0.06	0.08	0.07	0.10
SFA	5.03	5.17	4.94	5.43	6.02	7.32
UFA	5.02	4.41	3.19	8.98	8.71	13.22
CP	151.0	141.2	150.4	146.5	150.7	146.8
SDF	3.0	46.5	15.0	16.5	12.5	10.0
IDF	100.0	133.5	125.0	121.0	111.5	112.5
TDF	103.0	180.0	139.5	137.0	123.5	122.0
NDF	99.3	110.3	118.3	116.4	107.7	115.0
ADF	56.3	62.8	82.8	29.6	68.7	32.0
Hemicellulose	43.0	47.5	35.5	86.8	39.0	83.0

^a^*DRB* defatted rice bran, *SBP* sugar beet pulp, *RH* rice hull, *CGM* corn germ meal, *SH* soybean hull, *WB* wheat bran

^b^*DM* dry matter, *EE* ether extract, *SFA* saturated fatty acid, *UFA* unsaturated fatty acid, *CP* crude protein, *SDF* soluble dietary fiber, *IDF* insoluble dietary fiber, *TDF* total dietary fiber, *NDF* neutral detergent fiber, *ADF* acid detergent fiber

### Experimental procedures

After the recovery period form surgery, pigs were fed one of the six diets for six 10-day periods including a 6-day dietary acclimation period, following by a 2-day fecal sample collection and a 2-day ileal digesta sample collection. Feces were collected by grab sampling on d 7 and 8 and stored at − 20 °C. On d 9 and 10, ileal digesta samples were collected into plastic bags attached to the open cannula by cable ties. Digesta collection lasted for 9 h daily beginning at 08:00 h as described by Stein et al. [16]. The bags were removed whenever they were filled with digesta or at least every 30 min and then stored at − 20 °C to prevent bacterial degradation. At the end of the experiment, frozen fecal and digesta samples were thawed at room temperature, and then mixed by pig and period, sub-sampled and lyophilized in a vacuum-freeze dryer (Tofflon Freezing Drying Systems, Minhang District, Shanghai, China). Each dietary subsample was collected each week throughout the experimental periods and in the feed mill during mixing. At the end of the experiment, dietary samples were pooled and subsampled for chemical analyses. Before chemical analyses, diets, dried fecal samples and dried digesta subsamples were finely ground through a 1-mm screen and thoroughly mixed.

### Analytical methods

For Exp. 1 and Exp. 2, all diets, feces and digesta were analyzed for dry matter (DM; method 930.15) [18], and crude fat (ether extract; EE) [19]. Crude protein (CP) in all diets was measured using method 984.13 [18]. Neutral detergent fiber and acid detergent fiber (ADF) were measured using filter bags and fiber analyzer equipment (Fibre Analyzer, Ankom Technology, Macedon, NY) following a modification of the procedures described by Van Soest et al. [20]. The NDF was analyzed using heat stable α-amylase and sodium sulfite without correction for insoluble ash. Analysis of chromium (Cr) in all diets, feces and digesta utilized using a polarized Zeeman Atomic Absorption Spectrometer (Hitachi Z2000, Tokyo, Japan) after nitric acid-perchloric acid wet ash sample preparation. The fatty acid profiles of all diets, feces and digesta were determined using Gas Chromatography (6890 Series, Agilent Technologies, Wilmington, DE) according to the procedures of Sukhija and Palmquist [21] with the slight modification of using undecanoic acid (C11:0) (Sigma-Aldrich, St. Louis, MO) as the internal standard. Fatty acids were converted to fatty acid methyl esters using methanolic HCl. Aliquots of 1 μL were injected into a capillary column (60 m × 250 mm × 250 nm, DB-23, Agilent) in which cyanopropyl methyl silicone was the stationary phase. The column oven temperature was programmed with a 1:20 split. Injector and detector temperatures were maintained at 260 °C and 270 °C, respectively. Nitrogen was the carrier gas at a flow rate of 2 mL/min. All chemical analyses were conducted in duplicate.

### Calculations

In Exp. 1 and Exp. 2, the endogenous loss of fat and each fatty acid was calculated for ileal digesta and total enteral feces from pigs fed the fat-free diets in using the following equation that to determine the endogenous loss of nitrogen (N) for each amino acid (AA) as described by Stein et al. [22]:

$$ {\displaystyle \begin{array}{l}{\mathrm{IF}}_{\mathrm{end}}=\left[{\mathrm{F}}_{\mathrm{d}}\times \left({\mathrm{Cr}}_{\mathrm{f}}/{\mathrm{Cr}}_{\mathrm{d}}\right)\right],\\ {}{\mathrm{IF}\mathrm{A}}_{\mathrm{end}}=\left[{\mathrm{F}\mathrm{A}}_{\mathrm{d}}\times \left({\mathrm{Cr}}_{\mathrm{f}}/{\mathrm{Cr}}_{\mathrm{d}}\right)\right]\end{array}} $$

IF_end_ and IFA_end_ are the endogenous losses of fat and a fatty acid (g/kg of DM intake; DMI) in ileal digesta or total enteral feces, respectively. F_d_ and FA_d_ represent the concentration of fat and fatty acid in the ileal digesta or feces from pigs fed the fat-free diet, respectively. Cr_d_ represents the concentrations of Cr in the ileal digesta or feces. The Cr content in fat-free diets is represented by Cr_f_.

### Statistical analyses

In Exp. 1, Orthogonal polynomial contrasts were used to detect the linear and quadratic responses of increasing NDF content in diets. Data in Exp. 2 were analyzed by ANOVA using the MIXED procedure of SAS (SAS Institute Inc., Cary, NC). The individual pig was the experiment unit for all analyses. In Exp. 1, the model consisted of NDF content as the fixed effect and pig and period as random effects. In Exp. 2, the model contained fiber-rich ingredients as the fixed effect and pig and period as random effects. The significant differences among treatments in Exp. 2 were detected by Tukey’s Multiple Range Test. In Exp. 1 and Exp. 2, the REPEATED statement was used to model the effect of period using individual pig as the subject from which repeated observations were recorded [23]. The normality of the data was detected using the UNIVARIATE procedure of SAS (SAS Institute Inc., Cary, NC) and no outliers were detected in either Exp. 1 or Exp. 2. The LSMEANS in SAS was chosen to calculate means. The respective contributions in percentages of the upper gut and hindgut to endogenous losses of fat and fatty acids for each dietary treatment in Exp.1 and Exp.2 were tested by *t*-test. An alpha level of *P* <  0.05 was set as the criterion for statistical significance in both experiments.

## Results

### Exp. 1: effect of dietary fiber content on endogenous loss of fat

The amounts (g/kg DMI) of endogenous losses of fat and fatty acids and the fatty acids as a percent of total fatty acids (% of TFA) at the end of ileum and throughout the intestinal tract in growing pigs fed different fiber content diets are summarized in Tables 5 and 6, respectively. Based on the percentage of TFA in Tables 5 and 6, the profile of endogenous loss of fatty acids did not change as dietary NDF increased. The C16:0, C18:0, C18:1 and C18:2 were the main components of endogenous loss of fatty acids at the end of ileum and throughout the entire intestinal tract. Moreover, these fatty acids, fat, total unsaturated fatty acids (UFA) and total saturated fatty acids (SFA) increased linearly (*P* <  0.01) as the NDF content of diets increased. The endogenous losses of fat as well as C16:0 and C18:0 throughout the entire intestinal tract in growing pigs increased quadratically as NDF content of diets increased. The endogenous loss of fat increased from 0.71 to 3.14 g/kg of dry matter intake (DMI) and 0.56 to 8.21 g/kg DMI at the end of ileum and throughout the entire intestinal tract in growing pigs, respectively.

**Table 5:** Effect of fiber content on amount (g/kg DMI) of losses of endogenous fat and fatty acids, as well as the percentages of losses of individual fatty acids relative to total fatty acids (% of TFA) at the end of ileum of growing pigs (Exp. 1, DM basis)

Items^b^	Neutral detergent fiber content of diets^a^												SEM*	*P*-value^*^	
	0 g/kg		40 g/kg		80 g/kg		120 g/kg		160 g/kg		200 g/kg			Linear	Quadratic
	g/kg DMI	% of TFA	g/kg DMI	% of TFA	g/kg DMI	% of TFA	g/kg DMI	% of TFA	g/kg DMI	% of TFA	g/kg DMI	% of TFA			
EE	0.71	–	1.05	–	1.86	–	2.06	–	2.59	–	3.14	–	0.27	< 0.01	0.96
C12:0	0.02	2.94	0.01	0.66	0.02	0.77	0.02	0.68	0.02	0.41	0.02	0.32	0.00	0.37	0.72
C14:0	0.02	2.94	0.03	1.97	0.04	1.53	0.05	1.71	0.06	1.24	0.07	1.13	0.01	< 0.01	0.55
C15:0	0.00	0.00	0.01	0.66	0.01	0.38	0.01	0.34	0.02	0.41	0.02	0.32	0.00	< 0.01	0.84
C16:0	0.21	30.88	0.43	28.29	0.78	29.89	0.87	29.69	1.30	26.86	1.60	25.93	0.11	< 0.01	0.51
C17:0	0.01	1.47	0.02	1.32	0.03	1.15	0.03	1.02	0.05	1.03	0.06	0.97	0.00	< 0.01	0.34
C18:0	0.12	17.65	0.23	15.13	0.41	15.71	0.44	15.02	0.65	13.43	0.80	12.97	0.06	< 0.01	0.53
C18:1	0.09	13.24	0.26	17.11	0.51	19.54	0.58	19.80	0.95	19.63	1.21	19.61	0.06	< 0.01	0.12
C18:2	0.06	8.82	0.25	16.45	0.44	16.86	0.49	16.72	1.06	21.90	1.47	23.82	0.12	< 0.01	0.03
C18:3	0.00	0.00	0.05	3.29	0.09	3.45	0.10	3.41	0.24	4.96	0.32	5.19	0.03	< 0.01	0.05
C20:0	0.01	1.47	0.02	1.32	0.03	1.15	0.04	1.37	0.06	1.24	0.07	1.13	0.00	< 0.01	0.05
C20:1	0.02	2.94	0.02	1.32	0.02	0.77	0.03	1.02	0.04	0.83	0.05	0.81	0.00	< 0.01	0.05
C21:0	0.01	1.47	0.01	0.66	0.01	0.38	0.00	0.00	0.01	0.21	0.01	0.16	0.00	0.23	0.50
C20:3	0.00	0.00	0.00	0.00	0.00	0.00	0.00	0.00	0.01	0.21	0.01	0.16	0.00	< 0.01	0.23
C20:4	0.01	1.47	0.01	0.66	0.01	0.38	0.00	0.00	0.01	0.21	0.01	0.16	0.00	0.97	0.04
C22:0	0.02	2.94	0.03	1.97	0.04	1.53	0.05	1.71	0.08	1.65	0.09	1.46	0.01	< 0.01	0.20
C22:1	0.01	1.47	0.01	0.66	0.01	0.38	0.02	0.68	0.02	0.41	0.02	0.32	0.00	0.02	0.80
C23:0	0.01	1.47	0.02	1.32	0.03	1.15	0.03	1.02	0.04	0.83	0.06	0.97	0.00	< 0.01	0.49
C24:0	0.04	5.88	0.07	4.61	0.10	3.83	0.12	4.10	0.16	3.31	0.21	3.40	0.02	< 0.01	0.48
C22:6	0.01	1.47	0.02	1.32	0.02	0.77	0.04	1.37	0.04	0.83	0.06	0.97	0.20	0.05	0.70
C24:1	0.01	1.47	0.02	1.32	0.01	0.38	0.01	0.34	0.02	0.41	0.01	0.16	0.00	0.47	0.47
SFA	0.47	69.12	0.88	57.89	1.50	57.47	1.66	56.66	2.45	50.62	3.01	48.78	0.19	< 0.01	0.47
UFA	0.21	30.88	0.64	42.11	1.11	42.53	1.27	43.34	2.39	49.38	3.16	51.22	0.19	< 0.01	0.02

^a^*DMI* Dry matter intake, *TFA* Total fatty acids;

^b^*EE* ether extract, *SFA* saturated fatty acid, *UFA* unsaturated fatty acid

^*^SEM and *P*-value all represent SEM and *P*-value of the amount (g/kg DMI) of endogenous losses of fat and fatty acids only

**Table 6:** Effect of fiber content on amount (g/kg DMI) of losses of endogenous fat and fatty acids, as well as percentages of fatty acids relative to total fatty acids (% of TFA) over the total intestinal tract of growing pigs (Exp. 1, DM basis)

Items^b^	Neutral detergent fiber content of diets^a^												SEM*	*P*-value^*^	
	0 g/kg		40 g/kg		80 g/kg		120 g/kg		160 g/kg		200 g/kg			Linear	Quadratic
	g/kg DMI	% of TFA	g/kg DMI	% of TFA	g/kg DMI	% of TFA	g/kg DMI	% of TFA	g/kg DMI	% of TFA	g/kg DMI	% of TFA			
EE	0.56	–	1.63	–	4.79	–	5.89	–	7.14	–	8.21	–	0.41	< 0.01	0.02
C12:0	0.00	0.00	0.01	0.50	0.01	0.22	0.02	0.37	0.02	0.30	0.03	0.44	0.00	< 0.01	0.46
C13:0	0.00	0.00	0.00	0.00	0.01	0.22	0.01	0.19	0.01	0.15	0.02	0.29	0.00	< 0.01	0.94
C14:0	0.02	2.17	0.05	2.48	0.09	1.98	0.11	2.06	0.13	1.98	0.16	2.33	0.01	< 0.01	0.55
C15:0	0.06	6.52	0.13	6.44	0.24	5.29	0.33	6.18	0.38	5.79	0.43	6.26	0.03	< 0.01	0.03
C16:0	0.32	34.78	0.60	29.70	1.14	25.11	1.30	24.34	1.57	23.93	1.63	23.73	0.08	< 0.01	0.01
C17:0	0.03	3.26	0.08	3.96	0.15	3.30	0.19	3.56	0.21	3.20	0.22	3.20	0.02	< 0.01	0.02
C18:0	0.33	35.87	0.83	41.09	2.17	47.80	2.60	48.69	3.20	48.78	3.20	46.58	0.23	< 0.01	0.01
C18:1	0.03	3.26	0.08	3.96	0.18	3.96	0.20	3.75	0.28	4.27	0.34	4.95	0.02	< 0.01	0.78
C18:2	0.01	1.09	0.07	3.47	0.21	4.63	0.21	3.93	0.28	4.27	0.36	5.24	0.05	< 0.01	0.70
C18:3	0.00	0.00	0.02	0.99	0.05	1.10	0.05	0.94	0.08	1.22	0.08	1.16	0.01	< 0.01	0.40
C20:0	0.05	5.43	0.05	2.48	0.09	1.98	0.10	1.87	0.10	1.52	0.10	1.46	0.01	< 0.01	0.09
C20:1	0.00	0.00	0.00	0.00	0.01	0.22	0.01	0.19	0.02	0.30	0.02	0.29	0.00	< 0.01	0.94
C21:0	0.00	0.00	0.00	0.00	0.00	0.00	0.01	0.19	0.01	0.15	0.02	0.29	0.00	< 0.01	0.92
C20:3	0.00	0.00	0.00	0.00	0.00	0.00	0.00	0.00	0.01	0.15	0.00.	0.00	0.00	0.03	0.22
C20:4	0.00	0.00	0.00	0.00	0.00	0.00	0.01	0.19	0.02	0.30	0.01	0.15	0.00	0.04	0.36
C22:0	0.02	2.17	0.03	1.49	0.06	1.32	0.06	1.12	0.07	1.07	0.07	1.02	0.01	< 0.01	0.19
C22:1	0.00	0.00	0.01	0.50	0.01	0.22	0.01	0.19	0.01	0.15	0.01	0.15	0.00	0.03	0.12
C23:0	0.00	0.00	0.01	0.50	0.01	0.22	0.02	0.37	0.01	0.15	0.02	0.29	0.01	0.04	0.73
C24:0	0.03	3.26	0.04	1.98	0.07	1.54	0.07	1.31	0.09	1.37	0.09	1.31	0.01	< 0.01	0.36
C24:1	0.02	2.17	0.01	0.50	0.04	0.88	0.03	0.56	0.06	0.91	0.06	0.87	0.02	< 0.01	0.85
SFA	0.86	93.48	1.83	90.59	4.04	88.99	4.82	90.26	5.80	88.41	5.99	87.19	0.36	< 0.01	< 0.01
UFA	0.08	6.52	0.19	9.41	0.50	11.01	0.52	9.74	0.76	11.59	0.88	12.81	0.10	< 0.01	0.67

^a^*DMI* Dry matter intake, *TFA* Total fatty acids

^b^*EE* ether extract, *SFA* saturated fatty acid, *UFA* unsaturated fatty acid

^*^SEM and *P*-value all represent SEM and *P*-value of the amount (g/kg DMI) of endogenous losses of fat and fatty acids only

The endogenous loss of total SFA is a little different from that of total UFA at the end of ileum, whereas the endogenous loss of total SFA is much greater than that of total UFA over the entire intestinal tract. On the whole, the endogenous losses of fat, individual SFA and total SFA over the entire intestinal tract were much greater than those at the end of ileum, however, the endogenous losses of individual UFA and total UFA were lower over the entire intestinal tract.

The respective contributions in percentages of losses in the upper gut and hindgut to endogenous EE and fatty acids are summarized in Table 7. The ELF occurred in the hindgut except for growing pigs were fed 0 and 4% NDF in their diets. The endogenous losses of C16:0 and UFA were primarily in the upper gut and the endogenous losses of C18:0 were greatest in the hindgut.

**Table 7:** The respective contributions in percentages of the upper gut (UG) and the hindgut (HG) to endogenous losses of fat and fatty acids in growing pigs fed diets that differed in content of NDF. (Exp. 1, DM basis)^a^

Items^b^, %	Neutral detergent fiber content of diets, g/kg																	
	0^c^			40			80			120			160			200		
	UG	HG	SE	UG	HG	SE	UG	HG	SE	UG	HG	SE	UG	HG	SE	UG	HG	SE
EE	127	−27	4	64	36	2	39	61	1	35	65	2	36	64	2	38	62	1
C16:0	66	34	2	72	28	1	68	32	3	67	33	2	83	17	1	98	2	1
C18:0	36	64	1	28	72	1	19	81	2	17	83	3	20	80	3	25	75	1
C18:1	300	−200	9	325	−225	5	283	− 183	6	290	− 190	6	339	− 239	7	356	−256	6
C18:2	600	− 500	8	357	−257	5	209	−109	4	233	− 133	3	379	−279	8	408	− 308	4
C18:3	–	–		250	−150	7	180	−80	4	200	−100	5	300	−200	5	400	−300	6
SFA	55	45	1	48^NS^	52^NS^	1	37	63	2	34	66	2	42	58	2	50^NS^	50^NS^	1
UFA	350	−250	8	337	−237	7	222	− 122	5	244	− 144	4	314	−214	6	359	− 259	5

^a^In this table, the respective contribution in percentages of upper gut and hindgut were compared by T-test in each dietary treatment. No superscript indicated that there was a significant difference in the respective contribution in percentages of the upper gut and hindgut each dietary treatment. The superscript “NS” represented that there was no significant difference. The SE represented the standard error of the results of upper gut or hindgut in each experimental treatment

^b^*EE* ether extract, *SFA* saturated fatty acid, *UFA* unsaturated fatty acid

^c^*UG* upper gut, *HG* hindgut, *SE* standard error

### Exp. 2: effect of different fiber-rich ingredients on endogenous loss of fat

The amounts (g/kg DMI) of endogenous losses of fat and fatty acids and the percentage of TFA at the end of ileum in growing pigs fed diets in which fiber-rich ingredients supplements were DRB, SBP, RH, CGM, SH or WB are presented in Table 8. The results showed that ELF at the end of ileum in growing pigs fed CGM or WB diets were greater (*P* <  0.01) than for pigs fed the RH, DRB, SBP and SH diets. The profile of endogenous losses of fatty acids changed to a slight degree at the end of ileum that the endogenous losses of UFA (particularly C18:1 and C18:2) in growing pigs fed CGM or WB diets were greater (*P* <  0.01) than that for the other four diets and the greatest (*P* <  0.01) endogenous losses of SFA (particularly C18:0) was in the growing pigs fed the RH diet compared to the other five diets. The endogenous loss of C16:0 at the end of ileum in growing pigs fed the RH, CGM or WB diets were greater (*P* <  0.01) than for the other three diets. In general, results indicated that the endogenous losses of fat or fatty acids at the end of ileum was greater in growing pigs fed RH, CGM or WB diets.

**Table 8:** Effect of fiber-rich ingredients on amounts (g/kg DMI) of losses of endogenous fat and fatty acids and fatty acids as a percent of total fatty acids (% of TFA) at the end of ileum of growing pigs (Exp. 2, DM basis)

Items^e^	Fiber-rich ingredients of diets^d^												SEM^*^	*P*-value^*^
	250 g/kg DRB		270 g/kg SBP		145 g/kg RH		250 g/kg CGM		170 g/kg SH		280 g/kg WB			
	g/kg DMI	% of TFA	g/kg DMI	% of TFA	g/kg DMI	% of TFA	g/kg DMI	% of TFA	g/kg DMI	% of TFA	g/kg DMI	% of TFA		
EE	1.34^c^	–	2.58^b^	–	4.60^a^	–	3.50^ab^	–	1.54^c^	–	4.17^ab^	–	0.46	< 0.01
C12:0	0.02	1.10	0.03	1.35	0.07	1.71	0.03	0.60	0.02	0.75	0.03	0.61	0.02	0.16
C14:0	0.03^b^	1.66	0.05^b^	2.25	0.10^a^	2.44	0.04^b^	0.80	0.04^b^	1.50	0.04^b^	0.81	0.01	< 0.01
C15:0	0.01^b^	0.55	0.02^b^	0.90	0.09^a^	2.20	0.01^b^	0.20	0.01^b^	0.38	0.01^b^	0.20	0.02	0.03
C16:0	0.44^bc^	24.31	0.73^b^	32.88	1.26^a^	30.73	1.11^a^	22.33	0.80^b^	30.08	1.17^a^	23.83	0.13	< 0.01
C17:0	0.04	2.21	0.01	0.45	0.04	0.98	0.02	0.40	0.03	1.13	0.02	0.41	0.01	0.21
C18:0	0.18^c^	9.94	0.16^c^	7.21	1.02^a^	24.88	0.27^bc^	5.43	0.40^b^	15.04	0.23^bc^	4.68	0.19	0.04
C18:1	0.42^bc^	23.20	0.27^c^	12.16	0.50^bc^	12.20	1.32^a^	26.56	0.52^b^	19.55	0.98^a^	19.96	0.07	< 0.01
C18:2	0.24^c^	13.26	0.54^b^	24.32	0.29^c^	7.07	1.67^a^	33.60	0.45^bc^	16.92	1.97^a^	40.12	0.16	< 0.01
C18:3	0.01^c^	0.55	0.05^bc^	2.25	0.01^c^	0.24	0.07^bc^	1.41	0.09^ab^	3.38	0.12^a^	2.44	0.01	< 0.01
C20:0	0.02	1.10	0.01	0.45	0.06	1.46	0.05	1.01	0.03	1.13	0.02	0.41	0.01	0.20
C20:1	0.02	1.10	0.02	0.90	0.04	0.98	0.04	0.80	0.02	0.75	0.06	1.22	0.01	0.07
C21:0	0.01	0.55	0.01	0.45	0.03	0.73	0.01	0.20	0	0.00	0.01	0.20	0.01	0.10
C20:4	0.02^b^	1.10	0.02^b^	0.90	0.05^a^	1.22	0.02^b^	0.40	0.00^b^	0.00	0.01^b^	0.20	0.01	< 0.01
C22:0	0.06	3.31	0.04	1.80	0.06	1.46	0.04	0.80	0.04	1.50	0.04	0.81	0.01	0.54
C22:1	0.03	1.66	0.03	1.35	0.04	0.98	0.02	0.40	0.01	0.38	0.01	0.20	0.01	0.06
C23:0	0.02	1.10	0.03	1.35	0.03	0.73	0.02	0.40	0.03	1.13	0.02	0.41	0.01	0.24
C24:0	0.15^b^	8.29	0.14^b^	6.31	0.28^a^	6.83	0.10^b^	2.01	0.11^b^	4.14	0.11^b^	2.24	0.03	< 0.01
C22:6	0.04	2.21	0.04	1.80	0.06	1.46	0.04	0.80	0.04	1.50	0.01	0.20	0.03	0.89
C24:1	0.05	2.76	0.02	0.90	0.07	1.71	0.09	1.81	0.02	0.75	0.05	1.02	0.02	0.10
SFA	0.98^c^	54.14	1.23^bc^	55.41	3.04^a^	74.15	1.70^b^	34.21	1.51^bc^	56.77	1.70^b^	34.62	0.39	0.02
UFA	0.83^c^	45.86	0.99^bc^	44.59	1.06^b^	25.85	3.27^a^	65.79	1.15^b^	43.23	3.21^a^	65.38	0.24	< 0.01

^a-c^Within a row, means followed by the same or no superscript letter are not different (*P* > 0.05)

^d^*DRB* defatted rice bran, *SBP* sugar beet pulp, *RH* rice hull, *CGM* corn germ meal, *SH* soybean hull, *WB* wheat bran, *DMI* Dry matter intake, *TFA* Total fatty acids

^e^*EE* ether extract, *SFA* saturated fatty acid, *UFA* unsaturated fatty acid

^*^SEM and *P*-value all represent SEM and *P*-value of the amount (g/kg DMI) of endogenous losses of fat and fatty acids only

The amounts (g/kg DMI) of endogenous losses of fat and fatty acids and the percentage of TFA over the entire intestinal tract in growing pigs fed the six above-mentioned common ingredients diets are summarized in Table 9. The results indicated that ELF was the greatest (*P* <  0.01) in growing pigs fed CGM or WB diets; however, the ELF over the entire intestinal tract was not the highest in growing pigs fed the RH diet among the six fiber-rich ingredients and the result was opposite that at the end of ileum. The endogenous loss of C16:0 was greater (*P* <  0.01) in growing pigs fed the RH, CGM or WB diets. The greastest (*P* <  0.01) endogenous loss of C18:0 was in growing pigs fed the CGM and WB diets. The endogenous loss of SFA was the greatest (*P* <  0.01) in growing pigs fed the CGM, WB and RH diets. The least (*P* <  0.01) endogenous losses of fat, C16:0, C18:0, and SFA were in growing pigs fed the DRB diets. Overall, the endogenous losses of fat, C16:0, C18:0 and SFA over the entire intestinal tract were much greater (*P* <  0.01) in growing pigs fed the CGM and WB diets, whereas the lowest (*P* <  0.01) values were in growing pigs fed the DRB diet.

**Table 9:** Effect of fiber-rich ingredients on amounts (g/kg DMI) of losses of endogenous fat and fatty acids and fatty acids as a percent of total fatty acids (% of TFA) over the total intestinal tract of growing pigs (Exp. 2, DM basis)

Items^f^	Fiber-rich ingredients of diets^e^												SEM^*^	*P*-value^*^
	250 g/kg DRB		270 g/kg SBP		145 g/kg RH		250 g/kg CGM		170 g/kg SH		280 g/kg WB			
	g/kg DMI	% of TFA	g/kg DMI	% of TFA	g/kg DMI	% of TFA	g/kg DMI	% of TFA	g/kg DMI	% of TFA	g/kg DMI	% of TFA		
EE	1.99^c^	–	5.11^b^	–	5.34^b^	–	7.23^a^	–	4.88^b^	–	7.88^a^	–	0.82	< 0.01
C12:0	0.01^b^	0.65	0.02^a^	0.58	0.02^a^	0.41	0.02^b^	0.31	0.01^b^	0.23	0.01^b^	0.17	0.00	< 0.01
C13:0	0.00^b^	0.00	0.01^a^	0.29	0.01^ab^	0.20	0.01^a^	0.15	0.01^ab^	0.23	0.01^ab^	0.17	0.00	0.03
C14:0	0.03^c^	1.94	0.13^b^	3.76	0.09^bc^	1.84	0.13^b^	2.00	0.10^b^	2.27	0.08^bc^	1.38	0.02	< 0.01
C15:0	0.12^c^	7.74	0.40^a^	11.56	0.20^bc^	4.10	0.30^b^	4.62	0.26^b^	5.91	0.20^bc^	3.45	0.03	< 0.01
C16:0	0.43^c^	27.74	1.20^b^	34.68	1.47^a^	30.12	1.42^a^	21.85	1.10^b^	25.00	1.40^a^	24.18	0.16	< 0.01
C17:0	0.06	3.87	0.14	4.05	0.13	2.66	0.14	2.15	0.15	3.41	0.14	2.42	0.03	0.25
C18:0	0.48^d^	30.97	1.06^c^	30.64	2.18^b^	44.67	3.74^a^	57.54	2.12^b^	48.18	3.16^a^	54.58	0.41	< 0.01
C18:1	0.14^b^	9.03	0.11^b^	3.18	0.17^ab^	3.48	0.24^a^	3.69	0.17^ab^	3.86	0.18^ab^	3.11	0.02	0.01
C18:2	0.06^b^	3.87	0.11^b^	3.18	0.06^b^	1.23	0.15^ab^	2.31	0.17^ab^	3.86	0.26^a^	4.49	0.03	< 0.01
C18:3	0.01^b^	0.65	0.01^b^	0.29	0.02^b^	0.41	0.03^ab^	0.46	0.05^a^	1.14	0.03^ab^	0.52	0.01	< 0.01
C20:0	0.05^b^	3.23	0.05^b^	1.45	0.21^a^	4.30	0.12^b^	1.85	0.07^b^	1.59	0.10^b^	1.73	0.03	< 0.01
C20:1	0.01^b^	0.65	0.01^ab^	0.29	0.01^ab^	0.20	0.01^ab^	0.15	0.01^ab^	0.23	0.02^a^	0.35	0.00	0.03
C22:0	0.03^b^	1.94	0.05^ab^	1.45	0.10^a^	2.05	0.06^ab^	0.92	0.05^ab^	1.14	0.05^ab^	0.86	0.01	0.09
C22:1	0.01	0.65	0.01	0.29	0.02	0.41	0.01	0.15	0.01	0.23	0.01	0.17	0.00	0.27
C23:0	0.01	0.65	0.03	0.87	0.01	0.20	0.01	0.15	0.01	0.23	0.02	0.35	0.01	0.42
C24:0	0.06	3.87	0.08	2.31	0.09	1.84	0.06	0.92	0.07	1.59	0.07	1.21	0.02	0.66
C24:1	0.04	2.58	0.04	1.16	0.09	1.84	0.05	0.77	0.04	0.91	0.05	0.86	0.02	0.62
SFA	1.28^c^	82.58	3.17^bc^	91.62	4.51^b^	92.42	6.01^a^	92.46	3.95^b^	89.77	5.25^a^	90.50	0.64	< 0.01
UFA	0.27	17.42	0.29	8.38	0.37	7.58	0.49	7.54	0.45	10.23	0.57	9.50	0.08	0.09

^a-d^Within a row, means followed by the same or no superscript letter are not different (*P* > 0.05)

^e^*DRB* defatted rice bran, *SBP* sugar beet pulp, *RH* rice hull, *CGM* corn germ meal, *SH* soybean hull, *WB* wheat bran, *DMI* Dry matter intake, *TFA* Total fatty acids

^f^*EE* ether extract, *SFA* saturated fatty acid, *UFA* unsaturated fatty acid

^*^SEM and *P*-value all represent SEM and *P*-value of the amount (g/kg DMI) of endogenous losses of fat and fatty acids only

Overall comparisons revealed that the endogenous losses of C18:1, C18:2 and UFA over the entire intestinal tract of growing pigs fed the six common fiber-rich ingredients diets decreased compared with that at the end of ileum, whereas the endogenous losses of fat, C16:0, C18:0 and SFA were greater. The respective contributions in percentages of the upper gut and hindgut to endogenous losses of EE and fatty acids are summarized in Table 10. The contribution in percentages of ELF of upper gut was greater than of hindgut in growing pigs fed DRB and RH diets, while the contribution of upper gut and hindgut were equal in growing pigs fed SBP, CGM and WB diets. The endogenous losses of C16:0 and UFA occurred in the upper gut and the endogenous losses of C18:0 were mainly in the hindgut.

**Table 10:** The respective contribution in percentages of the upper gut (UG) and the hindgut (HG) to endogenous losses of fat and fatty acids in growing pigs fed diets that differed in fiber-rich ingredients (Exp. 2, DM basis)^a^

Items^b^, %	Fiber-rich ingredients of diets^c^																	
	250 g/kg DRB			270 g/kg SBP			145 g/kg RH			250 g/kg CGM			170 g/kg SH			280 g/kg WB		
	UG	HG	SE	UG	HG	SE	UG	HG	SE	UG	HG	SE	UG	HG	SE	UG	HG	SE
EE	67	33	2	50^NS^	50^NS^	2	86	14	3	48^NS^	52^NS^	2	32	68	1	53^NS^	47^NS^	2
C16:0	102	−2	3	61	39	2	86	14	2	78	22	1	73	27	2	84	16	3
C18:0	38	62	1	15	85	1	47	53	2	7	93	2	19	81	1	7	93	1
C18:1	300	−200	6	245	−145	5	294	− 194	4	550	− 450	8	306	−206	7	544	− 444	5
C18:2	400	−300	6	491	− 391	7	483	− 383	5	1113	− 1013	6	265	− 165	5	758	−658	8
C18:3	100	0	5	500	− 400	4	50^NS^	50^NS^	3	233	−133	3	180	−80	3	400	−300	6
SFA	77	23	2	39	61	1	67	33	2	28	72	1	38	62	2	32	68	2
UFA	307	− 207	6	341	−241	5	286	− 186	5	667	− 567	6	255	−155	6	583	− 483	6

^a^In this table, the respective contribution in percentages of upper gut and hindgut were compared by T-test in each dietary treatment. No superscript indicated that there was a significant difference in the respective contribution in percentages of the upper gut and hindgut each dietary treatment. The superscript “NS” represented that there was no significant difference. The SE represented the standard error of the results of upper gut or hindgut in each experimental treatment

^b^*EE* ether extract, *SFA* saturated fatty acid, *UFA* unsaturated fatty acid

^c^*DRB* defatted rice bran, *SBP* sugar beet pulp, *RH* rice hull, *CGM* corn germ meal, *SH* soybean hull, *WB* wheat bran, *UG* upper gut, *HG* hindgut, *SE* standard error

## Discussion

### Exp. 1: effect of dietary fiber content on endogenous loss of fat

Previous reports indicated that ELF varied from 1.37 to 22.4 g/kg DMI over the entire intestinal tract in pigs fed extracted fat [8, 10, 11, 24, 25] and from 2.0 to 12.08 g/kg DMI in pigs fed intact fat [10, 11, 26, 27]. Those studies all used the graded levels of fat regression method to estimate ELF values. The significant differences among the above studies may be the reason that the lowest content of dietary fat and the range of fat content in diets were different. The key factor for accurate determination of regression coefficients is a broad gradient range [28]. On the other hand, other chemical components in diets may also affect ELF values [14, 29, 30]. However, reports on the effect of the non-fat chemical component on the ELF was very limited. Kil et al. [11] and Kim et al. [31] reported that ELF values are lower in pigs fed extracted fat than intact fat which diets contained higher content of fiber, suggesting that the higher fiber content in diets results in greater ELF values.

In the current study, the results indicated that the ELF value at the end of ileum and over the entire intestinal tract increased linearly (*P* <  0.01) as the NDF content in the diets increased. Those results confirm results reported by Kil et al. [11] and Kim et al. [31]. Except for the pigs fed 0 and 40 g/kg NDF diets, the values obtained from the current study were within the ranges reported by others [8, 10, 11, 24–27]. This was linked to the low fiber content (0 and 40 g/kg) in the diets which were far below the fiber content of normal diets for growing pigs. The higher content of fiber in diets could reduce the absorption of dietary fat and reabsorption of endogenous fat before the end of ileum, which would result in much higher concentration of ELF at the end of ileum and over the entire intestinal tract [14]. In addition, the higher content of fiber may also promote the fermentation of fiber that could produce volatile fatty acids (VFA), and stimulate excretion of bile acids, growth of the microbial population and desquamation of epithelial cells [32–34]. Those materials could be released into the feces as a portion of the fat content of feces in the hindgut of pigs and an increase of ELF in the hindgut [32–34]. This may be one reason for greater ELF values over the entire intestinal tract compared to the end of ileum in the present study. The result was similar to that reported by Kil et al. [11] who found the ELF value at the end of the ileum to be less than that for the entire intestine (7.27 vs 12.08 g/kg DMI) in pigs fed intact fat, while the ELF value was not different between the two collection sites (3.28 vs 3.77 g/kg DMI) in pigs fed extracted fat. This phenomenon confirmed an effect of fiber on ELF in the hindgut of growing pigs.

In addition to the above factors, another factor that increased the endogenous loss of fatty acids as NDF content of diets increased in growing pigs was the *de novo* synthesis of fatty acids by the microbial population in the gut indicating intestinal fermentation of fiber [1, 14, 35]. Particularly, the endogenous losses of fat, individual SFA and total SFA over the entire intestinal tract were greater than that at the end of ileum, whereas the endogenous loss of individual UFA and total UFA was just the opposite in the current study. These results were consistent with those reported by Jørgensen et al. [8]. The difference may be due to hydrogenation of UFA to SFA by the gut microflora [29, 36]. Besides, the result that endogenous losses of fat, as well as C16:0 and C18:0 over the entire intestinal tract in growing pigs increased quadratically as NDF content increased in diets of pigs. Thus, the greatest endogenous losses of fat, and C16:0 and C18:0 occurs in growing pigs fed diets containing 160 or 200 g/kg NDF, but may not increase the endogenous loss of fat, C16:0 and C18:0 over the entire intestinal tract in growing pigs fed diets containing more than 160 or 200 g/kg NDF. This conjecture needs to be confirmed by feeding greater amounts of NDF (e.g. 240, 280 and 320 g/kg) in diets of growing pigs.

Fiber could promote hindgut fermentation and microbial growth, leading to the higher contribution of the hindgut to ELF in growing pigs as dietary NDF increased, except as noted for pigs fed 0 and 4% NDF in their diets [1, 35]. The greater contribution of endogenous losses of C16:0 in the upper gut may due to desquamation of epithelial cells and biliary secretions that were less affected by fermentation of fiber in the hindgut [32]. However, the hindgut fermentation of fiber and hydrogenation of C18:1 and C18:2 increases endogenous losses of C18:0 in the hindgut [29, 32].

### Exp. 2: effect of different fiber-rich ingredients on endogenous loss of fat

Apart from the concentration of fiber in diets of growing pigs, the effect of fiber on digestion and absorption of nutrients is associated closely with the functional and structural characteristics of fiber [37–39]. In the Exp. 1 of the present study, the endogenous losses of fat and fatty acids increased along with increases in amounts of SH. In contrast, Kil et al. [11] reported that a purified NDF (isolated cellulose) had little depressive effect on digestibility of fat and ELF. The discrepancy was possibly the result of the different characteristics of fiber between SH and purified NDF. The purified NDF was primarily cellulose that is not easily digested, and may have little stimulatory effect on microbial growth and production of ELF [11]. Similarly, Gao et al. [38] reported that the addition of carboxymethylcellulose sodium (CMC) to pig diets had no effect on the ATTD of fat, while the addition of inulin (INU) increased ATTD of fat. Those results also contributed to the discrepancy of fiber characteristics between the two sources of fiber. The CMC has high viscosity and low fermentation, which has a less stimulatory effect on microbial growth, whereas INU has low viscosity and high fermentation potential to increase intestinal fermentation and the synthesis of fatty acids. Thus, available results indicate that fiber type could have a significant effect on the microbial population in the gut and affect the endogenous loss of fat and the digestibility of fat [11, 38, 40]. Therefore, we selected six common fiber-rich ingredients with different fiber characteristic to determine the effects on endogenous loss of fat and fatty acids in growing pigs, and to provide data for optimizing digestibility and energy supplying when fiber-rich ingredients were used in diets of growing pig.

Results of the present study revealed that there was a significant difference in the endogenous loss of fat and fatty acids at the end of ileum in pigs fed DRB, SBP, RH, CGM, SH or WB diets. This may be related strongly to the characteristics and physicochemical structure of fiber (e.g., solubility, viscosity and fermentability) [38, 39, 41, 42]. The ELF values at the end of ileum in growing pigs fed RH, CGM or WB diets were the highest. This may be due to the higher content of hemicellulose in CGM and WB diets and the higher concentration of lignin in the RH diet, while concentrations of cellulose were higher in DRB and SH diets [43] and the soluble dietary fiber (SDF) content was the greatest in the SBP diet [44]. The SDF was fermented more easily, rapidly and completely [40], while insoluble dietary fiber (IDF) would prolong degradation and fermentation [14, 45, 46] resulting in higher ELF value than SDF. Hemicellulose and lignin are more readily fermentable than cellulose and stimulated microbial growth and the synthesis of fatty acids to yield a higher content of ELF [11, 47–49]. The higher endogenous losses of C16:0 at the end of ileum in pigs fed RH, CGM and WB diet may be a result of the role of hemicellulose to promote the synthesis of C16:0 and lipolysis *in vivo* [1, 39] or due to effects to facilitate the microbial growth, desquamation of epithelial cells and biliary secretions with a higher C16:0 content [32–34]. The endogenous loss of C18:0 was the greatest at the end of ileum in pigs fed the RH diet, which indicated that lignin may promote the synthesis of C18:0 [1] or stimulate microbial growth, the desquamation of epithelial cells or biliary secretions *in vivo* that have greater concentrations of C18:0 [32–34]. The endogenous losses of C18:1 and C18:2 were the greatest in pigs fed CGM and WB diets. This may due to effects of hemicellulose on gut microbes, the desquamation of epithelial cells and biliary secretions that increase C18:1 and C18:2. But, it may also be connected to the fatty acid content of body storage in pigs before the experiment, because C18:1 and C18:2 are not synthesized *in vivo* in pigs.

However, the endogenous losses of fat and C18:0 were the greatest over the entire intestinal tract of pigs fed CGM or WB diets only, but values were not so high in pigs fed the RH diet. The result indicated that the RH diet with higher lignin content may have a stimulatory effect on ELF before the end of ileum primarily and have little effect on ELF in the hindgut. The endogenous losses of fat, C16:0 and C18:0 and SFA over the entire intestinal tract in pigs fed the DRB diet were the lowest may due to DRB having less effects on endogenous loss of fat and SFA in the hindgut compared with effects of SBP and SH in the diet, but evidence for this requires further study. Similar to results from Exp.1, the endogenous loss of C18:1 and C18:2 and UFA over the entire intestinal tract of growing pigs fed the six common fiber-rich ingredients diets was lower than that at the end of ileum, while the endogenous losses of C16:0 and C18:0 and SFA were much higher. The hydrogenation of UFA in hindgut may explain those results [29, 36].

Therefore, different fiber-rich ingredients have different effects on the endogenous losses of fat and fatty acids. Considering the requirement of fat and fatty acids for pigs, the effects of dietary fiber ingredients on the endogenous losses of fat and fatty acids should be considered when developing feed formulations.

## Conclusions

In conclusion, the profile of endogenous losses of fatty acids did not change as dietary NDF increased in growing pigs. The C16:0, C18:0, C18:1 and C18:2 were the main components of endogenous loss of fatty acids at the end of ileum and over the entire intestinal tract and they increased significantly with the increases in NDF content of diets for pigs. The ELF occurred in the hindgut except for growing pigs fed diets with 0 and 4% NDF. The endogenous losses of C16:0 and UFA were primarily in the upper gut while greater endogenous losses of C18:0 occurred in the hindgut. The endogenous losses of fat or fatty acids at the end of ileum were greater in growing pigs fed RH, CGM and WB diets. The higher endogenous losses of fat, C16:0 and C18:0, and SFA over the entire tract of pigs fed CGM or WB diets, while those values were the lowest in growing pigs fed the DRB diet. In addition, the endogenous losses of individual or total UFA was less over the entire intestinal tract than at the end of ileum of growing pigs fed fiber diets, and the higher endogenous losses of fat, as well as individual or total SFA occurred over the entire intestinal tract. Therefore, different fiber content or fiber-rich ingredients in pig diets have different influence on the endogenous losses of fat and fatty acids. Considering the dietary requirements of pigs for fat and fatty acids, careful attention must be paid to avoid the endogenous losses of fat and fatty acids when fiber ingredients are used in diets of pigs.

## Abbreviations

AA: Amino acid
ADF: Acid detergent fiber
ATTD: Apparent total tract digestibility
CGM: Corn germ meal
CMC: Carboxymethylcellulose sodium
CP: Crude protein
Cr: Chromium
DM: Dry matter
DMI: Dry matter intake
DRB: Defatted rice bran
EE: Ether extract
ELF: Endogenous loss of fat
HG: Hindgut
IDF: Insoluble dietary fiber
INU: Inulin
N: Nitrogen
NDF: Neutral detergent fiber
RH: Rice hull
SBP: Sugar beet pulp
SDF: Soluble dietary fiber
SFA: Saturated fatty acid
SH: Soybean hull
TFA: Total fatty acids
TTTD: True total tract digestibility
UFA: Unsaturated fatty acid
UG: Upper gut
VFA: Volatile fatty acid
WB: Wheat bran
